# Silent Ischemic Heart Disease and Pericardial Fat Volume in HIV-Infected Patients: A Case-Control Myocardial Perfusion Scintigraphy Study

**DOI:** 10.1371/journal.pone.0072066

**Published:** 2013-08-14

**Authors:** Ulrik S. Kristoffersen, Anne-Mette Lebech, Niels Wiinberg, Claus L. Petersen, Philip Hasbak, Henrik Gutte, Gorm B. Jensen, Anne Mette F. Hag, Rasmus S. Ripa, Andreas Kjaer

**Affiliations:** 1 Department of Clinical Physiology, Nuclear Medicine and PET, Rigshospitalet, Copenhagen, Denmark; 2 Cluster for Molecular Imaging, University of Copenhagen, Copenhagen, Denmark; 3 Department of Infectious Diseases, Hvidovre University Hospital, Copenhagen, Denmark; 4 Department of Clinical Physiology and Nuclear Medicine, Frederiksberg University Hospital, Copenhagen, Denmark; 5 Department of Cardiology, Hvidovre University Hospital, Copenhagen, Denmark; 6 Copenhagen City Heart Study, Copenhagen University Hospital Bispebjerg, Department of Occupational Medicine, Copenhagen, Denmark; University of New South Wales, Australia

## Abstract

**Objectives:**

to determine the prevalence of asymptomatic ischemic heart disease (IHD) in HIV patients by myocardial perfusion scintigraphy (MPS) and to determine the value of coronary artery calcium score (CACS), carotid intima-media thickness (cIMT) and pericardial fat volume as screening tools for detection of IHD in subjects with HIV.

**Background:**

Patients with HIV seem prone to early development of IHD.

**Methods:**

105 consecutive HIV patients (mean age 47.4 years; mean duration of HIV 12.3 years; mean CD4+ cell count 636×10^6^/L; all receiving antiretroviral therapy) and 105 controls matched for age, gender and smoking status, without history of IHD were recruited. MPS, CACS, cIMT, pericardial fat volume, and cardiovascular risk scores were measured.

**Results:**

HIV patients demonstrated higher prevalence of perfusion defects than controls (18% vs. 0%; p<0.001) despite similar risk scores. Of HIV patients with perfusion defects, 42% had a CACS = 0. CACS and cIMT were similar in HIV patients and controls. HIV patients on average had 35% increased pericardial fat volume and increased concentration of biomarkers of atherosclerosis in the blood. HIV patients with myocardial perfusion defects had increased pericardial fat volume compared with HIV patients without perfusion defects (314±43 vs. 189±12 mL; p<0.001).

**Conclusions:**

HIV patients had an increased prevalence of silent IHD compared to controls as demonstrated by MPS. The finding was strongly associated with pericardial fat volume, whereas cardiovascular risk scores, cIMT and CACS seem less useful as screening tools for detection of myocardial perfusion defects in HIV patients.

## Introduction

Since the introduction of combination antiretroviral therapy (cART), mortality has decreased substantially in the HIV infected population [1], [2]. In this setting, cardiovascular disease (CVD) has shown to be a leading cause of morbidity and mortality in patients with HIV.

Since effective treatments are available to reduce the risk of CVD in patients at risk, early identification of subclinical coronary atherosclerosis is of importance. However, the probable different mechanisms behind development of CVD in HIV patients may limit the usefulness of standard screening tools for CVD.

Myocardial perfusion defects demonstrated by myocardial perfusion scintigraphy (MPS) are one of the most prognostic measures that predict cardiovascular events and death in the general population [3]. Accordingly, it would be of interest to study the prevalence of such myocardial perfusion defects also in HIV infected patients without known CVD.

In the general population carotid intima-media thickness (cIMT) correlates well with the presence of ischemic heart disease (IHD) and screening by coronary artery calcium score (CACS) is an accepted measure of atherosclerotic burden [4]. This may not be true if accelerated atherosclerosis with soft plaques is the major cause of vascular changes in HIV infected patients [5], [6]. Additionally, pericardial fat volume has recently been associated with both coronary artery calcification [7] and non-calcified coronary plaque burden [8], [9]. This association might also be different in HIV patients, especially since lipodystrophy may influence fat distribution differently than in HIV negative patients.

The aim of our study was to determine the prevalence of asymptomatic IHD in HIV patients well-managed on cART by means of MPS. In addition, all HIV patients and controls were screened by CACS, cIMT, pericardial fat volume and biomarkers of cardiovascular risk in order to elucidate which screening methods for CVD could be used in HIV infected patients.

## Methods

### Ethics Statement

Written informed consent was obtained from all participants and the study was approved by the Scientific Ethical Committee of the Capital Region of Denmark (KF H-C-2008-060).

### Design

A total of 105 consecutive HIV patients were prospectively recruited at routine visits at the outpatient clinic at Department of Infectious Diseases, Hvidovre University Hospital between September 2008 and July 2010. Inclusion criteria were i) HIV infected, ii) age 18–70 years and iii) receiving cART>12 months. Exclusion criteria were i) any symptoms or signs suggesting ischemic heart disease. ii) pregnancy, risk of pregnancy or lactation, iii) known heart disease, iv) statin treatment, v) thyrotoxicosis, vi) allergy to contrast media, vii) renal impairment or viii) claustrophobia. However, no subjects were excluded on basis of statin treatment or renal impairment.

Every HIV patient, a control subject was matched 1∶1 for age, gender and smoking status. This was done in the following way: for each HIV patient 3 age, gender and smoking matched controls were identified in the Copenhagen City Heart Study database [10] and were contacted. The first positive responder was included. None of the control subjects had any symptoms or signs suggesting ischemic heart disease. We did not find any difference between included and non-included controls.

All patients and control subjects had an MPS, CACS, pericardial fat volume measurement, cIMT measurement and a blood sample analysis performed after inclusion in the trial.

### Myocardial perfusion scintigraphy

MPS was conducted in accordance with procedural guidelines of the European Association of Nuclear Medicine. Stress testing was preferentially performed with bicycle ergometer exercise using a standard Bruce protocol (at least 85% of the predicted maximal heart rate was achieved). Stress studies were obtained in all patients. Images were acquired 60 minutes after tracer injection of 600–800 MBq of Tc-99 m sestamibi. Gated single photon emission computed tomography myocardial perfusion images were obtained with a Philips Precedence 16P SPECT/CT scanner (Philips Medical Systems, the Netherlands). A simultaneous CT was used for attenuation correction. Myocardial perfusion images were analyzed using Cedars-Sinai Autoquant 7.2 (Los Angeles, California). All examinations were analyzed by very experienced readers, blinded for HIV status. All examinations were adjudicated as normal or with significant perfusion defect as a consensus reading between two readers. This adjudication was based on all available information; scintigrams with and without attenuation correction, extent and position of any defect, left ventricular volumes and regional wall motion abnormalities.

### Coronary artery calcium scoring

CAC scanning was performed using a Philips Precedence 16P SPECT/CT scanner (Philips, Medical Systems, the Netherlands). Quantification of CACS was done ad modum Agatston as previously described [11].

### Pericardial adipose tissue measurement

Pericardial fat volume was measured on the previously obtained CT-images of the heart. A region of interest was traced on the boundaries of the pericardial layers in every 10 mm. Volume analysis software (Philips, Medical Systems, the Netherlands) was used to discern fat from other tissues with a threshold of −190 to −30 Hounsfield units. The volume was the sum of all voxels containing fat. Pericardial fat includes both epicardial (located within the pericardium) and paracardial fat (located superficial to the pericardium) [12].

### Carotid intima media thickness

cIMT was determined using a high-resolution ultrasound Doppler system 128 XP/10c (Acuson, GE Medical Systems Information Technologies, Inc., Milwaukee, WI, US) with a linear 7–10 MHz transducer and automatic IMT-measuring tool of image evaluation software (Math Resolutions, v. 20.1 METRIS, Argenteuil, France), as previously described [13].

### Blood sample analysis

Plasma lipids and intercellular adhesion molecule-1 (sICAM-1), soluble vascular cell adhesion molecule-1 (sVCAM-1), matrix metallopeptidase 9 (MMP9), tissue-type plasminogen activator inhibitor-1 (tPAI-1) and high-sensitivity C-reactive protein (hs-CRP) were measured as previously described [14]. Endothelin was measured with an enzyme-linked immunosorbent assay kit (Biomedica, Eching, Germany) that measures physiologically active Endothelin peptide (1–21). Lower detection limit was 0.02 fmol/mL, and the intra-assay and interassay CV's were 4% and 6%, respectively.

### Risk score and metabolic syndrome

The 10-year risk of cardiovascular events (Framingham risk score) [15] as well as the HIV specific DAD 5 year cardiovascular risk-assessment score [16] was calculated according to the definitions. Presence of metabolic syndrome was determined according to the International Diabetes Federation definition. In brief, this includes central obesity based on ethnicity specific waist circumference plus any two of the following: 1) raised triglycerides, reduced HDL-cholesterol, 3) raised blood pressure og 4) raised fasting plasma glucose. For further details please see reference [17].

### Data and Statistics

With a sample size of 90 matched pairs, a power of 80% for detection of a difference in prevalence of MPD of 8% vs. 0% in the two groups at an alpha = 0.05 was obtained in our one-to-one matched design. Data are presented as mean ± standard error of mean. For comparison between HIV patients and matched controls, categorical variables were compared with related samples McNemar's test and means of continuous variables were compared with paired t-tests. Adjusted p-values for continous variables were calculated using a random effects linear regression model. For comparison between HIV patients with and without perfusion defects, two sample t-test or Fisher's exact test were used for continuous and categorical variables, respectively. Adjusted p-values were calculated using a linear regression model. Binary logistic regression (with stepwise elimination of least significant covariate to test for independent association) was used to test for associations between test result (presence of perfusion defect) and background and examination parameters. Biomarker concentrations were log transformed prior to statistical analysis to obtain normal distribution but are presented untransformed. In some cases 95% confidence intervals (CI) are stated. Statistical analyses were performed using SPSS analysis software version 20.0 (IBM Software Group, New York, USA). A p-value <0.05 was considered significant.

## Results

### Characteristics of participants

Basic characteristics of the HIV (n = 105) and control (n = 105) populations are presented in table 1. At time of inclusion, 90% of the HIV patients had undetectable viral load (≤40 copies/mL). In the remaining patients the median viral load was 73 copies per mL (range 41–713). In the 11 patients with viral load ≥40 copies/mL only 3 patients had detectable load at two separate HIV RNA measurements done around the time of having the cardiac assessment done.

**Table 1:** Characteristics of the study groups.

	HIV patients		Controls		HIV patients vs. controls
	Mean	±SEM/(%)	Mean	±SEM/(%)	*p-value* *
Number	105		105		
Male gender	93	(89%)	93	(89%)	
Age (years)	47.4	±0.83	47.4	±0.83	
Tobacco users					
Current	39	(37%)	39	(37%)	
Former	11	(10%)	11	(10%)	
Hemodynamics					
Systolic blood pressure (mmHg)	130.7	±1.71	123.3	±1.48	*0.003*
Diastolic blood pressure (mmHg)	84.0	±1.12	81.9	±1.13	*0.2*
Pulse pressure (mmHg)	46.7	±1.44	41.4	±1.51	*0.015*
Mean arterial blood pressure (mmHg)	99.5	±1.16	95.7	±1.04	*0.024*
Heart rate (beats per minute)	80.1	±1.13	70.4	±1.46	*<0.001*
Anthropometry					
Height (cm)	178.4	±0.86	178.6	±0.79	*0.8*
Weight (kg)	78.8	±1.34	81.9	±1.22	*0.09*
Waist circumference (cm)	93.1	±1.02	92.9	±1.13	*0.9*
Body mass index (kg/m^2^)	24.7	±0.33	25.7	±0.35	*0.037*
Body surface area (m^2^)	1.97	±0.02	2.00	±0.02	*0.12*
Risk calculations					
Metabolic syndrome	40	(38%)	18	(17%)	*<0.001*
Framingham 10 yr CHD risk (%)	8.6	±0.6	7.7	±0.6	*0.09*
DAD 5 yr estimated risk (%)	3.9	±0.4	−	−	
Plasma values					
Total cholesterol (mmol/L)	5.85	±0.11	5.17	±0.10	*<0.001*
LDL-cholesterol (mmol/L)	3.60	±0.10	3.27	±0.09	*0.019*
HDL-cholesterol (mmol/L)	1.34	±0.04	1.39	±0.04	*0.4*
Triglycerides (mmol/L)	2.17	±0.18	1.14	±0.06	*<0.001*
Glucose (mmol/L)	5.63	±0.07	5.29	±0.10	*0.006*
HIV parameters					
CD4 cell count (10^6^/L)	636	±25			
CD4 nadir (10^6^/L)	171	±11			
HIV duration (years)	12.3	±0.64			
ART duration (years)	8.9	±0.41			
HIV RNA <40 copies/mL	94	(90%)			
≥2 NRTIs +1 NNRTI	65	(62%)			
≥2 NRTIs +≥1 PI	25	(24%)			
NRTI+NNRTI+PI	6	(6%)			
NRTI+PI+NNRTI+II	1	(1%)			
PI+NNRTI+II	1	(1%)			
PI+EI+II	2	(2%)			
Other	5	(5%)			

*) Paired t-test or Related sample McNemar's test when categorical variables. NRTI, nucleoside reverse transcriptase inhibitor.NNRTI, non-nucleoside reverse transcriptaseinhibitor. PI, proteaseinhibitor. II, integraseinhibitor. EI, entry inhibitor.CHD, coronary heart disease.

### Carotid intima media thickness, coronary calcium score, biomarkers and pericardial fat

Results of cardiovascular imaging by cIMT, CACS, pericardial fat volume and level of biomarkers are presented in table 2. HIV patients on average had a 35% higher amount of pericardial fat (p = 0.001) and increased levels of hsCRP, E-selectin, sVCAM-1, sICAM-1 and MMP9 (p<0.001). No differences were found in cIMT or CACS (dichotomized at 100) between HIV patients and controls. If dichotomizing CACS into 0 or above a borderline significance of p = 0.04 was found. If including only subjects without metabolic syndrome, 54 matched pairs were left for analyses. Among these pairs no difference was found between cIMT, CACS and tPAI1, Endothelin and sVCAM-1.

**Table 2:** Cardiovascular imaging and biomarker results.

	HIV patients		Controls		HIV patients vs. controls(adjusted p value^&^)
	Mean	±SEM/(%)	Mean	±SEM/(%)	*p-value* *
Carotis intima media thickness					
Maximum (mm)	0.659	±0.013	0.649	±0.012	*0.5 (0.4)*
Minimum (mm)	0.566	±0.011	0.559	±0.010	*0.7 (0.3)*
Mean (mm)	0.613	±0.011	0.604	±0.010	*0.6 (0.3)*
Coronary artery calcium score	54.3	±16.7	105.0	±40.4	*0.5 (0.8)*
* Median*	*0*		*0*		
0	66	(63%)	79	(75%)	*0.04^#^*
>0 and ≤100	27	(26%)	18	(17%)	
>100	12	(11%)	8	(8%)	*0.5* §
Pericardial fat volume (mL)	211	±13	156	±10	*<0.001 (0.7)*
Biomarkers of cardiovascular risk					
hsCRP (µg/mL)	7.70	±1.05	3.54	±0.48	*<0.001 (0.002)*
E-selectin (ng/mL)	33.2	±1.3	22.5	±1.2	*<0.001 (0.001)*
sVCAM-1 (ng/mL)	1,252	±24	1,147	±19	*0.001 (0.001)*
sICAM-1 (ng/mL)	168.7	±6.7	120.4	±4.2	*<0.001 (0.001)*
MMP9 (ng/mL)	125.6	±8.5	86.0	±4.2	*<0.001 (0.004)*
tPAI-1 (ng/mL)	65.9	±3.3	68.5	±3.8	*0.6 (0.04)*
Endothelin (ng/mL)	2.27	±0.21	2.15	±0.20	*0.7 (0.7)*
Myocardial perfusion defect	19	(18%)	0	(0%)	*<0.001*
Reversible	14	(13%)	0	(0%)	
Irreversible	5	(5%)	0	(0%)	

*) Paired t-test or related sample McNemar's test when categorical variables. hsCRP, high.sensitivity C-reactive protein. sVCAM-1, soluble vascular cell adhesion molecule-1. sICAM-1, intercellular adhesion molecule-1. MMP9, matrix metallopeptidase 9. tPAI-1, tissue-type plasminogen activator inhibitor-1.

#) Dichotomized in 0 or above;

§ ) dichotomized in ≤100 or >100;

&) adjusted for metabolic syndrome, systolic blood pressure, cholesterol, triglycerides and glucose using a random effects linear regression model.

### Myocardial perfusion defects

Perfusion defects were observed on MPS in 19 of the 105 HIV patients (18%) as opposed to none in the control group (p<0.001). The mean summed stress score in these patients were 7.8 with a mean extent of 10%. The patients with perfusion defects had a mean left ventricular ejection fraction of 52% with only one patient with an ejection fraction below 40%. Only one patient had a significant decrease in ejection fraction during stress compared to rest (from 51% to 43%). None of the patients had significant ECG changes or symptoms during the stress test performed as a maximal bicycle test (n = 18) or adenosine stress (n = 1). The heart rate reached the target of 85% of maximum pulse in all patients with bicycle stress test.

All 19 patients with perfusion defects were men. Nine (47%) had never smoked, 15 (79%) had CACS<100, and 8 (42%) had CACS = 0. Thirteen (68%) had metabolic syndrome.

HIV patients with perfusion defects on scintigrams had increased pericardial fat volume and waist circumference compared with HIV patients without defects on scintigrams (Table 3). No increased prevalence of perfusion defect was associated with cART containing versus not containing abacavir (20% vs. 16%, p = 0.6) or protease inhibitor (19% vs. 17%, p = 0.8). Also neither the traditional FRS nor the DAD 5 year risk differed between patients with and without perfusion defects (Table 3).

**Table 3:** Comparison of HIV patients with and without perfusion defects.

	Perfusion defects on MPS				
	Yes		No		
	N	(%)	N	(%)	*P-value* * *(adjusted p value^&^)*
	Mean	±SEM	Mean	±SEM	
Number	19		86		
Male gender	19	(100%)	74	(86%)	*0.12 (0.3)*
Age (years)	49.5	±2.1	46.9	±0.9	*0.2 (0.8)*
Ever tobacco users	10	(53%)	40	(46%)	*0.8 (0.04)*
Current	6	(32%)	33	(38%)	
Former	4	(21%)	7	(8%)	
Hemodynamics					
Systolic BP (mmHg)	131.8	±3.0	130.4	±2.0	*0.8 (0.4)*
Diastolic BP (mmHg)	85.2	±2.5	83.7	±1.3	*0.6 (0.8)*
Pulse pressure (mmHg)	46.6	±2.9	46.7	±1.6	*1.0 (0.8)*
Mean arterial BP (mmHg)	100.8	±2.3	99.3	±1.3	*0.6 (0.8)*
Heart rate (min^−1^)	77.7	±2.7	80.7	±1.2	*0.3 (0.2)*
Anthropometry					
Waist circumference (cm)	98.0	±1.8	92.0	±1.2	*0.022 (1.0)*
Body mass index (kg/m^2^)	25.8	±0.8	24.4	±0.4	*0.12 (0.8)*
Body surface area (m^2^)	2.05	±0.04	1.95	±0.02	*0.030 (0.4)*
Risk calculations					
Metabolic syndrome	13	(68%)	27	(31%)	*0.004 (0.04)*
Framingham 10 years risk	9.1	±1.1	8.5	±0.6	*0.7 (0.7)*
DAD 5yr estimated risk (%)	5.4	±1.2	3.6	±0.4	*0.3 (0.8)*
Plasma values					
Total cholesterol (mM)	5.89	±0.26	5.83	±0.12	*0.8 (0.6)*
LDL-cholesterol (mM)	1.29	±0.09	1.35	±0.05	*0.9 (0.4)*
HDL-cholesterol (mM)	3.62	±0.22	3.60	±0.12	*0.6 (0.6*
Triglycerides (mM)	2.34	±0.28	2.13	±0.22	*0.7 (0.5)*
Glucose (mM)	5.73	±0.17	5.61	±0.07	*0.5 (0.8)*
HIV parameters					
CD4 cell count (10^6^/L)	638	±52	635	±29	*1.0 (0.9)*
CD4 nadir (10^6^/L)	153	±21	175	±13	*0.5 (1.0)*
HIV duration (months)	136	±17	150	±9	*0.5 (0.3)*
ART duration (months)	101	±10	108	±6	*0.6 (0.4)*
HIV RNA <100 copies/mL	17	(89%)	80	(93%)	*0.6 (0.9)*
Carotis intima media thickness					
Maximum	0.67	±0.02	0.66	±0.02	*0.7 (0.4)*
Mean	0.63	±0.02	0.61	±0.01	*0.5 (0.4)*
Coronary artery calcium score	93	±42	46	±18	*0.3 (0.6)*
Median	12		0		
0	8	(42%)	58	(67%)	
>0 and ≤100	7	(37%)	20	(23%)	*0.10*
>100	4	(21%)	8	(9%)	
Pericardial fat volume	314	±43	189	±12	*<0.001 (0.05)*
Biomarkers					
hsCRP (mg/mL)	9.9	±3.4	7.2	±1.0	*0.6 (0.5)*
E-selectin (ng/mL)	37.0	±2.6	32.3	±1.5	*0.08 (0.3)*
sVCAM-1 (ng/mL)	1228	±48	1258	±28	*0.7 (0.7)*
sICAM-1 (ng/mL)	145	±11	174	±8	*0.07 (0.04)*
MMP9 (ng/mL)	162	±36	117	±6	*0.3 (0.2)*
tPAI-1 (ng/mL)	71.0	±6.8	64.8	±3.7	*0.3 (0.9)*
Endothelin (ng/mL)	2.26	±0.53	2.27	±0.23	*0.8 (0.6)*

*) two-sample t-test or Fisher's exact test. MPS, myocardial perfusion scintigraphy.BP, blood pressure.hsCRP, high-sensitivity C-reactive protein. sVCAM-1, soluble vascular cell adhesion molecule-1. sICAM-1, intercellular adhesion molecule-1. MMP9, matrix metallopeptidase 9.tPAI-1, tissue-type plasminogen activator inhibitor-1.

&) adjusted for metabolic syndrome, smoking status, age, gender, cholesterol, triglycerides, glucose and systolic blood pressure.

### Pericardial fat volume

In univariate binary logistic regression analysis, pericardial fat volume (odds ratio increase: 0.6% per mL [CI: 0.2–1.0%]; p = 0.002), waist circumference (odds ratio increase: 6% per centimeter [CI: 2–11%]; p = 0.026), body surface area (odds ratio increase: 189% per m^2^ [CI: 0.3–3,137%]; p = 0.034) and metabolic syndrome (odds ratio increase: 154% [CI: 45–263%]; p = 0.005) were all associated with presence of perfusion defect on MPS but age, smoking, cholesterol, systolic blood pressure og DAD 5 year risk were not. In multivariate analysis including all the above-mentioned parameters with backward elimination, only pericardial fat volume remained significant. The 8 HIV patients with a calcium score of 0 but perfusion defects identified on MPS had increased pericardial fat volume compared both with patients with no perfusion defect and a CACS of 0 (293±42 vs. 105±14 mL; p = 0.004) as well as with no perfusion defect regardless of CACS (293±42 vs. 189±12 mL; p = 0.013). In logistic regression analysis, pericardial fat volume was associated with presence of perfusion defect both when including only patients with a CACS of 0 in the model (odds ratio increase: 0.8% per mL [CI.: 0.1–1.4%]; p = 0.011) and when including all patients without perfusion defect regardless of CACS (odds ratio increase: 0.7% per mL [CI: 0.1–1.2%]; p = 0.021).

In univariate linear regression analyses pericardial fat volume in HIV-infected was associated with abdominal circumference (r = 0.57; p = 0.001); BMI (r = 0.46; p = 0.001); age (r = 0.35; p = 0.001); ART duration (r = 0.30; p = 0.002); HIV duration (r = 0.26; p = 0.007) and plasma glucose (r = 0.22; p = 0.02) whereas no correlation was found with CD4, CD4 nadir, HIV RNA, blood pressure, cholesterol or smoking. In a multivariate linear regression model with all the above-mentioned factors included and backward elimination of least significant factors only abdominal circumference, BMI and age remained in the final model (Table 4).

**Table 4:** Independent predictors of pericardial fat volume for HIV-patients and controls.

		β coefficient	SE	β coefficient(standardized)	p-value*
HIV-patients					
	Abdominal circumference (cm)	4.7	1.5	0.37	0.002
	BMI (kg/m^2^)	9.4	4.6	0.24	0.04
	Age (yr)	4.4	1.3	0.27	0.001
Controls					
	Abdominal circumference (cm)	4.1	1.0	0.47	0.002
	BMI (kg/m^2^)	6.5	2.9	0.23	0.03
	Age (yr)	1.7	0.9	0.14	0.06

* ) Multivariate linear regression model. The following additional parameters were included in the initial model but excluded during stepwise backwards elimination of least significant parameters: plasma glucose, blood pressure, cholesterol, smoking (cigarettes/day), HIV duration^#^, ART duration^#^, CD4^#^, CD4 nadir^#^, and HIV RNA^#^. #) Only analysis of HIV-patients.

In HIV-negative controls univariate linear regression analyses revealed that PFV was associated with abdominal circumference (r = 0.69; p = 0.001); BMI (r = 0.61; p = 0.001); age (r = 0.34; p = 0.001); and smoking (cigarettes per day; r = 0.22; p = 0.02) whereas no correlation was found with plasma glucose, blood pressure or cholesterol. In a multivariate linear regression model with all the above-mentioned factors included and backward elimination of least significant factors only abdominal circumference, BMI and age remained in final model (Table 4).

## Discussion

This is the first study to investigate silent ischemic heart disease by means of MPS in a group of optimally treated HIV patients and to compare it with an exact 1∶1 matched control group. The major finding was an increased prevalence of myocardial perfusion defects in HIV patients of 18% (95% CI: 12–27%) compared to 0% (95% CI: 0–3%) in HIV-negative controls.

Since an abnormal MPS is highly predictive of both fatal and non-fatal myocardial infarction [3] our finding fits into the observed increased rate of myocardial infarction in HIV patients [18]–[20]. An increased prevalence of subclinical coronary atherosclerosis in HIV patients has previously been found by investigating the degree of stenosis on CT-angiography or invasive coronary angiography [21], [22]. In several previous studies on non-HIV patients it has been shown that MPS is superior to degree of stenosis to predict future cardiovascular events and prognosis [23].

The fact that 18% of the HIV patients, all without known CVD, were found to have perfusion defects is potentially important, since intensive prophylactic treatment in these subjects may lead to fewer cardiovascular events. Accordingly, identification of valid screening tools was also a key aim of our study. Although much is known on how to screen the general population, the different pathogenesis in HIV patients is likely to make general screening algorithms less useful. In line with this, the applicability of Framingham risk score has been shown to be less predictive of cardiovascular event in HIV patients [16] probably since Framingham risk score is based on general life-long exposure to traditional risk factors whereas development of IHD in HIV patients is accelerated and induced by various factors related to HIV infection and treatment. Indeed, we found in the present study that the calculated Framingham risk score was the same in HIV patients with and without perfusion defects. We also calculated the DAD 5 year risk that is tailored to HIV patients [16]. However, also this risk score could not discriminate between HIV-infected with and without myocardial perfusion defect. In the general population CACS measurement correlates strongly with the area of the atherosclerotic plaque [24] and has an established value in coronary disease risk prediction [25]. However, we hypothesized that if HIV IHD is accelerated and caused by other mechanisms, calcification will be less likely and not reflect the risk of perfusion defects since calcification has no influence *per se* but is just a marker of plaque burden. In accordance with this idea, we did not find any clear association between CACS and presence of perfusion defects in HIV patients. Indeed, 42% (8 of 19) of the HIV patients with perfusion defects had a CACS of 0. In addition, the distribution of CACS in both controls and HIV patients were low and as predicted in normal subjects with an FRS of less than 5% [26].

Measurement of cIMT is associated with myocardial infarction and coronary artery disease in the general population [4]. Two studies found an independent association of HIV infection with greater cIMT [27], [28], whereas another study could not reproduce this [29]. In our study, we found no difference in cIMT between patients and controls, or between patients with and without perfusions defects. This could be due to either HIV patients having normal cIMT values or our healthy controls not being healthy. However, the values found in both groups are clearly at the level of healthy subject [30]. Measurement of circulating biomarkers may be used to risk stratify patients for IHD [31]. However, we could not find any differences in biomarkers of atherosclerosis between HIV patients with and without perfusion defects. This was expected based on our previous studies of these markers in HIV patients [32]. Most likely, HIV infection per se has such a large impact on the biomarkers investigated that any effect on the biomarkers from cardiovascular changes is likely to be overshadowed.

Thus, some of traditional screening tools in our study seem less useful in identifying HIV patients with myocardial perfusion defects.

Changes in fat metabolism and distribution occur and lipodystrophy is present in many HIV patients. Therefore, we also studied fat distribution both as abdominal circumference and by quantifying pericardial fat volume on CT since that latter has been associated with non-calcified plaque burden in HIV negatives [8], [9]. We found both pericardial fat volume and abdominal circumference to be higher in HIV patients with perfusions defects compared to those without. It should be noted that the amount of pericardial fat in controls was in accordance with previously reported values [33]. In an adjusted model tobacco use, metabolic syndrome, pericardial fat volume and sICAM-1 were associated with perfusion defect. However, in multivariate analysis, only pericardial fat volume remained independently associated with presence of perfusion defects. In agreement with this, epicardial adipose tissue was suggested to be a useful marker of CVD in a recent cross-sectional study of 876 HIV-infected patients although the study was limited by the lack of a control group [34]. Another very recent cross-sectional study of 213 HIV-infected patients indicated an association between high CACS and pericardial fat volume, but also this study was without a control group and further did not investigate presence of myocardial ischemia [35]. It is worth noting, that none of the HIV parameters or CRP carried any predictive value of perfusion defects pointing at HIV-related metabolic abnormalities rather than inflammation being the mechanism of increased perfusion defects seen in HIV.

This study is limited by the cross-sectional design making it impossible to predict the prognostic consequences of the positive MPS in these asymptomatic patients. Further, this study was not powered to detect possible effect of antiretroviral agents such as abacavir.

In conclusion, we observed that HIV patients have a high prevalence of silent ischemic heart disease that is not explainable by traditional risk factors but is strongly associated with pericardial fat volume. Prospective randomized studies should be undertaken to study the potential value identification and prophylactic treatment of these patients.
